# Hippocampal Neurogenesis, Depressive Disorders, and Antidepressant Therapy

**DOI:** 10.1155/2007/73754

**Published:** 2007-05-14

**Authors:** Eleni Paizanis, Michel Hamon, Laurence Lanfumey

**Affiliations:** ^1^INSERM, U677, 75013 Paris, France; ^2^Faculté de Médecine Pierre et Marie Curie, Université Pierre et Marie Curie-Paris 6, Site Pitié-Salpêtrière, IFR 70 des Neurosciences, UMR S677, 75013 Paris, France

## Abstract

There is a growing body of evidence that neural stem cells reside in the adult central nervous system where neurogenesis occurs throughout lifespan. Neurogenesis concerns mainly two areas in the brain: the subgranular zone of the dentate gyrus in the hippocampus and the subventricular zone, where it is controlled by several trophic factors and neuroactive molecules. Neurogenesis is involved in processes such as learning and memory and accumulating evidence implicates hippocampal neurogenesis in the physiopathology of depression. We herein review experimental and clinical data demonstrating that stress and antidepressant treatments affect neurogenesis in opposite direction in rodents. In particular, the stimulation of hippocampal neurogenesis by all types of antidepressant drugs supports the view that neuroplastic phenomena are involved in the physiopathology of depression and underlie—at least partly—antidepressant therapy.

## 1. NEUROGENESIS

Although some occasional reports of postnatal neurogenesis in mammals have been published during the first half of the twentieth century (see [1] for a review), it was only in the early 1960s that the first evidence of a postnatal neuronal proliferation was reported in various brain regions in adult rats, including the dentate gyrus of the hippocampus [2], the neocortex [3], and the olfactory bulb [4]. However, no consensus on this adult neurogenesis was reached at this period and these findings were somewhat forgotten for the next two decades mainly because of their apparent lack of functional relevance, and also because the definitive demonstration that the adult-generated cells were neurons rather than glia was not provided. It was only in the nineties that several technical developments allowed a clear-cut demonstration of neurogenesis in adult brain. It was then established that neural cell proliferation occurs throughout the lifespan in various species including rodents [5], monkeys [6], and humans [7], and is particularly important in two regions of the brain, the dentate gyrus of the hippocampus [5, 8] and the subventricular zone [9]. In the hippocampus, new granule cells are formed from progenitors located in the hilus of the dentate gyrus. During maturation and differentiation steps, newly generated cells enter the granule-cell layer, migrate through the layer towards the fissure, and get integrated into the basic circuitry of the hippocampus, notably through synaptic contacts with pyramidal neurons in the CA3 field [10, 11]. In the subventricular zone, neurogenesis gives rise to neurons that migrate through the rostral migratory stream and integrate the olfactory bulb as interneurons [12, 13].

**Figure 1:**
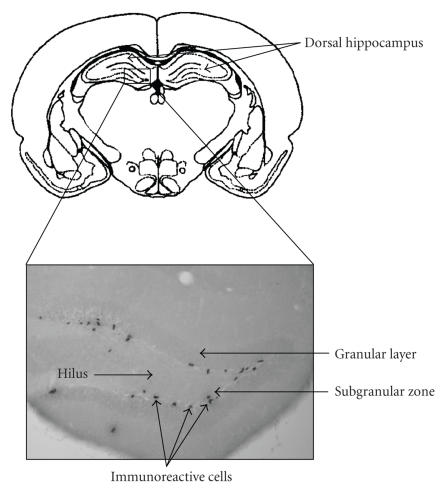
Photomicrograph of BrdU-positive cells in the subgranular zone of the dentate gyrus 2 hours after BrdU administration in an 8-week-old C57BL/6J mouse. Magnification: 100.

To label dividing cells, the earliest studies used [^3^H]-thymidine, which incorporates into replicating DNA during the S-phase of the cell cycle and can be detected by autoradiography [14]. An important technical improvement was the introduction of the synthetic thymidine analogue BrdU (5-bromo-3-deoxyuridine) that substitutes for thymidine in neosynthetized DNA of proliferating cells [15]. BrdU incorporated into DNA can then be easily visualized with immunocytochemical techniques using specific anti-BrdU antibodies. This technique allows quantitative analysis of proliferation, differentiation, and survival of newborn cells by varying the time interval between the pulse administration of BrdU and the sacrifice of animals [16–18]. The determination of the time and site of origin of newly generated cells in the CNS requires euthanasia shortly, generally between 1 and 3 hours, after the administration of BrdU, before newly born neurons have migrated out [19] (Figure 1).

For study of cell migration, immunocytochemical labeling has to be performed at various post-injection times, between 4 and 10 days, and finally, the fate and survival of the newly generated cells can be determined 21 days after BrdU injection, once migration has been achieved [5, 10, 20, 21].

Although DNA labeling by BrdU is currently the most commonly used method for studying adult neurogenesis, the potential toxic effect of this thymidine analogue should not be ignored as it might be a confounding factor in some experiments. This led to the use of other markers of the cell cycle, such as proliferating nuclear antigen (PCNA) and Ki-67, to analyze cell proliferation in situ [22]. PCNA, a cofactor of DNA polymerase, is expressed during the S-phase of cell cycle and quantification of both PCNA- and Ki-67-immunopositive cells has been shown to reliably reflect cellular proliferation, like BrdU labeling, in the adult DG [23].

In the rodent brain, approximately 9000 new neurons per day (i.e., 270 000 per month) are generated [24], and survive with a half-life of approximately 28 days [25]. This constitutive neurogenesis declines with age, as evidenced in rodents [26] and rhesus monkeys [27]. Although earliest studies on songbirds provided data in support of a functional role of adult neurogenesis in seasonal song learning [28], the possible functional significance of this process remains to be formally determined in mammals. However, the fact that hippocampal neurogenesis can be modulated by various factors including hormones, neurotransmitters, or environment suggests its real implication in physiological mechanisms and not its occurrence as a nonfunctional residual phenomenon in mammals [29]. In particular, glucocorticoids (including cortisol) have been shown to exert a negative influence that may account for the marked reduction in granule cell proliferation caused by stress [30], whereas, in contrast, antidepressant treatments markedly stimulate hippocampal neurogenesis [31]. The relevance of these data for pathophysiological mechanisms underlying depression is critically analyzed in the following section.

## 2. STRESS, GLUCOCORTICOIDS, AND NEUROGENESIS

Numerous studies have emphasized that stress can be the most significant causal agent, together with genetic vulnerability, in the etiology of depression. In addition, neurons in the hippocampal formation are among the most sensitive to the deleterious effects of stress. Consequently, stress-induced decrease in hippocampal neurogenesis might be an important feature associated with depression episodes.

Stress may be caused by any environmental change, whether internal or external, that disrupts the maintenance of homeostasis, and initiates a series of neuronal responses to prepare the organism to adapt to this new environmental challenge. Under environmental or psychological stressful conditions, neurons in the paraventricular nucleus (PVN) of the hypothalamus secrete corticotropin-releasing hormone/factor (CRH/CRF) and arginine-vasopressin (AVP), which in turn, stimulate the secretion of adrenocorticotropic hormone (ACTH) from the anterior pituitary gland. ACTH promotes the synthesis and the release of glucocorticoids from the adrenal cortex which allows the appropriate adaptation of the organism to stress, mainly through their vascular and metabolic effects [32].

The principal glucocorticoids are cortisol in humans and corticosterone in rodents. They both influence metabolism, cognitive processes, and emotions, especially fear and anxiety. To prevent deleterious effects of excessive levels of circulating glucocorticoids, the hypothalamic-pituitary-adrenal (HPA) stress axis is under tight control [32] through mineralocorticoid (MR) and glucocorticoid (GR) receptor negative feedback regulation [33]. Chronic stress frequently results in glucocorticoid and CRF hypersecretion associated with decreased sensitivity to glucocorticoid-mediated feedback inhibition. In vulnerable individuals, chronic stress may lead to excessively long lasting HPA responses that may precipitate psychopathologies such as anxiety and depression [34, 35].

Both basic and clinical studies have shown that stress can be associated with morphometric brain changes, neuronal atrophy, and decrease in the proliferation of progenitor cells in the hippocampal dentate gyrus. Whether these modifications really contribute to the development of depression is still a matter of debate [31, 36–39].

During the last decade, a series of reports indicated that major depression is frequently associated with significant atrophy within the hippocampus, which can persist for several years after remission from depression episodes [40–42]. In addition, prolonged depressions appeared to be associated with more severe atrophy [43]. Since the hippocampus plays a central role in learning and memory, these data suggested that such morphological alterations might be related to the cognitive deficits observed during depressive episodes [44, 45]. More recently, Stockmeier et al. [46] reported a reduction in both the average soma size of pyramidal neurons and neuropil, which may contribute to the volume retraction noted using fMRI in the hippocampus of patients with major depressive disorders. These morphometric alterations are most often attenuated or even reversed by antidepressants [46, 47].

Extensive preclinical investigations recently provided some keys toward understanding biological mechanisms causally related to hippocampus atrophy in severely depressed patients. In rodents, adrenal steroids were the first endogenous compounds to be identified as factors affecting hippocampal neurogenesis [48]. To date, adrenal steroids are well known to regulate both proliferation and differentiation of new neurons in the dentate gyrus [49]. In rats, a sustained increase in plasma corticosterone causes a decrease in neurogenesis while, reciprocally, adrenalectomy increases this process [50]. Indeed, removal of the adrenals accelerates neural cell proliferation and delays the death of newly formed neurons. Giving excess corticoids (e.g., corticosterone) has converse effects and consequently decreases the formation and survival of progenitor cells [51]. Treatment of adult male rats for 21 days with exogenous glucocorticoids has also a remodeling effect on dendrites in hippocampal neurons [52, 53].

In congruence with observations in depressed patients, both a reduction in hippocampal volume and a decrease in neurogenesis have been reported in subordinate tree shrews subjected to social interaction stress, which consists of a daily psychosocial conflict by introducing a naive animal into the cage of a socially experienced one [54, 55]. Changes in cell morphology, apical dendrite length, and branching of CA3 pyramidal cells were also observed in the same species under closely related experimental conditions [56]. Furthermore, chronic restraint stress for 21 days in rats led apical dendrites of CA3 pyramidal neurons to atrophy [57] and strongly reduced proliferation of dentate gyrus precursor cells [58]. Prenatal stress also decreases neurogenesis in the adult hippocampus along with increased anxiety-like behavior, hyperactivity of HPA axis, and reduced learning ability in rats [59] and exacerbated emotional behavior in rhesus monkeys [60]. On the other hand, inescapable stress leads to a reduction in neurogenesis that correlates with behavioral despair several days after exposure to stress in the learned helplessness model of depression [61]. Very recently, chronic mild stress, a validated paradigm to induce depression-like symptoms, has been shown to decrease survival (but not proliferation) of new born cells in adult rat hippocampus [62].

## 3. SEROTONIN, ANTIDEPRESSANTS, AND NEUROGENESIS

Serotonin, a key regulator of cell division, has been shown to modulate different processes such as neurogenesis, apoptosis, axon branching, and dendritogenesis during brain development [63]. This leads to propose for this neurotransmitter a critical role in the control of adult neural cell proliferation. In adult rats, the first study aimed at assessing the effect of 5-HT on neurogenesis was carried out using d,l-fenfluramine, which releases 5-HT throughout the central nervous system. Thus, Jacobs et al. [64] noted that d,l-fenfluramine increased cell division by two- to three-fold in the dentate gyrus. Subsequent studies confirmed the proliferating effect of 5-HT within the subgranular zone of the dentate gyrus [65], where both progenitor cells and a dense innervation by serotonergic fibers are observed [66]. Furthermore, a decrease in 5-HT content after either a lesion of serotonergic neurons by 5,7-dihydroxytryptamine (5,7-DHT) [67] or an inhibition of 5-HT synthesis by parachlorophenylalanine (PCPA) [67, 68] produced long-term deficits in the proliferation of hippocampal cells, and raphe grafts (which are enriched in 5-HT-producing neurons) reversed these deficits, very probably by replenishing endogenous 5-HT stores and restoring 5-HT functions [69].

The preferential involvement of 5-HT_1*A*_ receptors in the 5-HT effects on cell proliferation was first suggested by Jacobs et al. [64], who showed that d,l-fenfluramine -induced increase in neurogenesis was prevented by the selective 5-HT_1*A*_ receptor antagonist, WAY 100635. Later on, the promoting effect of 5-HT_1*A*_ receptor activation on hippocampal neurogenesis was confirmed by other groups. In particular, Santarelli et al. [70] noted that the 5-HT_1*A*_ receptor agonist, 8-OH-DPAT, caused an increase in cell proliferation in wild-type mice, but was ineffective in 5-HT_1*A*_ receptor knock-out mice, indicating that the action of 8-OH-DPAT was entirely mediated by 5-HT_1*A*_ receptors. However, other types of serotonergic receptors were also shown to be involved in the effects of serotonin on hippocampal cell proliferation. This is notably the case of 5-HT_2*A*_ and 5-HT_2*C*_ receptors whose activation by selective agonists enhanced neurogenesis in the rat dentate gyrus [65]. A simulatory effect was also noted with 5-HT_1*B*_ receptor agonists but only after 5-HT depletion [65]. Whether receptors of the 5-HT_4_, 5-HT_6_, and 5-HT_7_ types are also implicated in the regulation of hippocampal neurogenesis is still an open question to be addressed.

The clinical benefit of antidepressants that increase serotonergic neurotransmission such as selective serotonin reuptake inhibitors (SSRIs) drove several teams to analyze the effects of these drugs on cell proliferation and neurogenesis. A three-week systemic treatment with fluoxetine was first found to increase by 70 percent cell proliferation in the dentate gyrus in rodents [30, 31]. Because this effect was not observed in 5-HT_1*A*_ receptor knock-out mice, it could be inferred that 5-HT_1*A*_ receptor activation actually mediated fluoxetine-induced neurogenesis [71]. Several groups then confirmed that chronic, but not acute, antidepressant treatments exert a stimulatory influence on hippocampal neurogenesis [38, 61, 72]. Interestingly, all classes of antidepressant drugs tested so far, including NA and 5-HT reuptake inhibitors [30], atypical antidepressants such as tianeptine [54], electroconvulsive seizures, mood stabilizers such as lithium [31, 73], were shown to increase the proliferation and survival of new neurons in the dentate gyrus.

The lack of antidepressant-like effect of fluoxetine in x-irradiated mice, in which neurogenesis was abolished, led to the claim that clinical effectiveness of antidepressants is directly related to their promoting effect on hippocampal cell proliferation [71]. Interestingly, chronic treatments with CRH-R1 and V1b receptor antagonists, which are endowed with antidepressant-like properties in validated animal models [74, 75], also exerted a positive influence on hippocampal granule cell proliferation, thereby reversing the reduction in this process which had been caused by chronic mild stress [76]. Furthermore, the new antidepressant agomelatine also exerts a stimulatory influence on cell proliferation within the hippocampus. Chronic administration of this mixed MT1/MT2 melatonin receptor agonist and 5-HT_2*B*/2*C*_ receptor antagonist significantly increased the number of new born cells in the hippocampus of adult rats [77], and reversed the deficit in granule cell proliferation that had been induced at adult stage in rats born from a mother subjected to repeated stress during gestation [78]. In line with these observations, preliminary data from our laboratory showed that chronic treatment with fluoxetine or agomelatine compensated for the deficit in neurogenesis observed in transgenic GR-i mice (a murine model of depression, [79]), and raised this process up to the level observed in healthy paired wild-type mice [80]. These data are compatible with the idea that all types of antidepressant treatments apparently share the capacity to enhance cell proliferation and neurogenesis in the dentate gyrus of the hippocampus, thereby antagonizing the reduction in this process that has been regularly observed in validated animal models of depression (GR-i mice, learned heplessness, genetically helpless mice, chronic psychosocial stress, etc.) and is very likely occurring also in patients during a severe depression episode.

5-HT and corticotrope systems are closely cross-regulated under normal physiological conditions in mammals [81, 82] and their interactions are of particular relevance when considering pathological conditions such as depression, in which dysfunctioning of both systems has been consistently documented [83–86]. Although the exact mechanisms by which stress and glucocorticoids on the one hand, and antidepressants and serotonin on the other hand, affect neurogenesis have not been completely elucidated, evidence has been reported that modifications in the expression of brain-derived neurotrophic factor (BDNF) in the hippocampus might be part of the causal event [87]. BDNF is a major neurotrophic factor in brain, which plays key roles in the survival and guidance of neurons during development, and is required for the survival and normal functioning of neurons in the adult brain [88]. Decreased levels of BDNF in response to stress could lead to a loss of normal plasticity and also to damage and death of neurons. It is conceivable that the cell loss observed in depression could result from alterations in factors that control programmed cell death including cAMP response element binding (CREB) protein. Indeed, Dowlatshahi et al. [89] reported that CREB levels are decreased in the cerebral cortex of depressed patients. Conversely, several studies demonstrated that antidepressant treatment upregulates cAMP production, and, in turn, the CREB cascade including CREB-induced expression of BDNF [90]. Interestingly, upregulation of CREB and BDNF occurs not only in response to chronic treatment with various classes of antidepressant drugs, including NA and/or 5-HT reuptake inhibitors, but also after electroconvulsive seizures mimicking electroconvulsive therapy. Accordingly, it can be inferred that the cAMP-CREB cascade and BDNF are common postreceptor targets of both glucocorticoids and antidepressant treatments [70, 91] and thus very probably participate in associated neuroplastic phenomena.

## 4. CONCLUSION

The data summarized in this review highlight the involvement of hippocampal plasticity in physiopathological processes linked to mood disorders. Both corticotrope and serotonin systems have largely been involved in depressive symptoms and most of the effective antidepressant therapies are known to act through them. Interestingly, these two systems induce sustained modifications in adult hippocampal neurogenesis. However, much remains to be understood about the relations between cell proliferation, the hippocampus, and depression. Although hippocampal neurogenesis appears to be necessary for antidepressant drugs to alleviate depression-related behavioral deficits, it is probably not the case for the positive behavioral effects of environmental enrichment [92] and the antidepressant therapy using transcranial magnetic stimulation [93]. Accordingly, relationships between cell proliferation and antidepressant therapy are probably much more complex than originally claimed. However, the observation that cell proliferation parallels the effects of antidepressant drugs may lead to set up new strategies to treat depressive disorders. To this aim, elucidating the cellular and molecular mechanisms of action of antidepressants on neurogenesis is the further critical steps to be achieved.
